# The impact of anatomy variation on temperature based time of death estimation

**DOI:** 10.1007/s00414-023-03026-w

**Published:** 2023-07-03

**Authors:** Julia Ullrich, Martin Weiser, Jayant Shanmugam Subramaniam, Sebastian Schenkl, Holger Muggenthaler, Michael Hubig, Gita Mall

**Affiliations:** 1 Zuse Institute Berlin, Takustraße 7, 14195 Berlin, Germany; 2 Institute of Forensic Medicine, Jena University Hospital - Friedrich Schiller University Jena, Am Klinikum 1, 07747 Jena, Germany

**Keywords:** Finite element method, Temperature-based time of death estimation, Anatomical models, Corpse cooling, Sensitivities

## Abstract

Temperature-based time of death estimation using simulation methods such as the finite element method promise higher accuracy and broader applicability in nonstandard cooling scenarios than established phenomenological methods. Their accuracy depends crucially on the simulation model to capture the actual situation, which in turn hinges on the representation of the corpse’s anatomy in form of computational meshes as well as on the thermodynamic parameters. While inaccuracies in anatomy representation due to coarse mesh resolution are known to have a minor impact on the estimated time of death, the sensitivity with respect to larger differences in the anatomy has so far not been studied. We assess this sensitivity by comparing four independently generated and vastly different anatomical models in terms of the estimated time of death in an identical cooling scenario. In order to isolate the impact of shape variation, the models are scaled to a reference size, and the possible impact of measurement location variation is excluded explicitly by finding measurement locations leading to minimum deviations. The thus obtained lower bound on the impact of anatomy on the estimated time of death shows, that anatomy variations lead to deviations of at least 5–10%.

## Introduction

Time of death estimation (TDE) is an important topic in forensic medicine, since the results may lead to convictions or acquittals in homicide trials. In the short to medium time range of about 24 h after death, temperature based TDE (TTDE) yields more accurate results than other TDE methods [1, 2]. TTDE is based on the assumption that the body core temperature *T* monotonously decreases over time *t* post mortem for ambient temperatures sufficiently below normal body temperature. The function *T*(*t*) is then called the model curve. The time since death $t:=t_m-t_d$ is defined as the difference between time $t_m$ of core temperature measurements during cooling process and time of death (ToD) $t_d$, which is the start of cooling. Assuming the model curve is known for all times post mortem, we obtain the temperature $T_m=T(t)$ for the time since death *t*. Then, the inverse function $T^{-1}(T_m)$ provides an estimate for the time since and hence the time of death. In general, no analytic expression for $T^{-1}(T_m)$ exists, such that its value must be computed numerically.

**Table 1:** Symbols and their meanings used throughout the paper

Symbol	Meaning	Defined in
*c*	specific heat capacity of tissue	Eq. 1
$D_2$	root mean squared relative deviation	Eq. 6
$D_\infty$	maximum relative deviation	Eq. 7
$\Delta t(T_m)$	relative deviations	Eq. 4
$\gamma$	effective heat transfer coefficient	Eq. 2
$J_q^M(x^M_\textrm{ref})$	mean total deviation w.r.t. to $D_q$ for $q={2,\infty }$	Eq. 8
$\kappa$	heat conductivity of tissue	Eq. 1
*M*	set of anatomy models	p. 4
$\rho$	mass density of tissue	Eq. 1
*t*	time	p. 1
*T*	body temperature distribution	Eq. 1
$t_a$	start of considered cooling time interval	p. 6
$t_b$	end of considered cooling time interval	p. 6
$T_m$	temperature measurement	p. 6
$t_x^M(T_m)$	times of death for model *M* at measurement $T_m$	p. 6
*x*	spatial positions	p. 1
$x_i^M$	set of spatial positions $i=1,\ldots$	p. 7
$x^M_\textrm{opt}$	family of optimized positions in models *M*	p. 7
$x^M_\textrm{ref}$	family of reference positions in models *M*	p. 7

All TTDEs mainly differ in their approach to generate an accurate model curve *T*(*t*). Phenomenological methods use more or less specific formulas with parameters fitted in real cooling experiments. In forensic practice in Europe, the most often applied TTDE method is the phenomenological method by Marshall and Hoare with parameter definitions by Henßge (MHH) (see, e.g., [3–5]). Due to the ad hoc character of its model function, MHH has to use a “soft” parameter called correction factor tuning the body weight to apply MHH to non-standard cooling conditions such as clothing, convection, or body position. A drawback of this approach is that aspects like irradiation and changes in environmental temperature as well as anatomical factors like the actual height of the body are not captured by this method, leading to uncertainties in TDE.

The limitations of phenomenological models can be overcome by thermodynamical methods [2]. TTDE methods belonging to the class of such physics based models solve the heat transfer equation to obtain the model curve *T*(*t*). Here, the model curve results from a detailed simulation of physical processes, containing parameters like heat capacities, conductivities, corpse geometry, tissues and ambient conditions. Their accuracy is important for the TDE, having different impacts on the cooling curve [6]. With an appropriate parameter choice, the physical model can also capture nonstandard situations [7].

The heat transfer model can be solved numerically for *T*(*t*) by the finite element method (FEM). This approach has been investigated for use in TTDE in, e.g., [2, 7, 8]. A discretization strategy for implementing a cooling scenario combined with techniques from partial differential equations theory transform the initial and boundary value problem of heat transfer into a sequence of linear equation systems which can be solved numerically on a computer [9].

One of the main drawbacks in FEM is the increased effort in constructing a geometrical representation of the cooling bodies in the form of hexahedral or simplicial meshes. Up to now, the influence of how good these meshes match the actual bodies’ geometries in terms of the resulting estimated ToD *t* was not investigated systematically. Consequently, FEM approaches aim at representing the geometries with high fidelity. A step to reduce the effort was taken in [10]. Here, the data gained from computed tomography (CT) scans were semi-automatically segmented and meshed into an FE model, on which the actual cooling could be simulated and the model curve for TTDE could be obtained.

A sensitivity analysis was performed for several model parameters of the FEM for TTDE [6]. The impact of geometric fidelity with respect to mesh resolution had been investigated only for a single underlying geometry. Describing changes of the geometry by a limited number of anatomically meaningful parameters is difficult. This complicates the sensitivity analysis of TTDE methods with respect to the model geometry.

In forensic casework, the rectal temperature is used as a measure of the body core temperature of the corpse. There are different recommendations concerning the thermometer insertion depth. Henssge [11] recommends inserting the thermometer as deep as possible without using force, Madea [5, chapter 3] suggests an insertion depth of at least $8\,\text {cm}$. However, insertion depth is not determined exactly and relies much on practical and theoretical experiences in TTDE. Hence, the location of the measurement position is subject to variations. The sensitivity of estimated time of death with respect to measurement position has been investigated in a previous study [12].

The present study investigates the sensitivity of time of death with respect to anatomic variation. The simulation results for four independent and different FE meshes, each scaled to a similar body weight and length, are compared in terms of estimated time of death. The cooling curves are determined at defined measurement positions. Differences between the curves due to variations in these positions are minimized by optimizing the positions. The remaining difference is then due to the different anatomies and provides a lower bound for the sensitivity of estimated time of death with respect to the anatomy.

In Sect. 2, the methods and models used for the simulation are introduced. The thermodynamic heat equation for the FEM is presented. For simulation, the four full-body models used here and the simulation setup and parameters are described in detail. After selecting a reference position for each of the four anatomies, they are compared by evaluating the according temperature curves. For this purpose, we introduce a relative difference measure for quantifying the deviation of cooling curves. In Sect. 3, inter- and intra-anatomical deviations are described and the measurement positions are optimized across the models, leading to a lower bound for the impact of anatomies. Results for the inter- and intra-anatomical deviations before and after optimization are presented in Sect. 4, together with the evaluation of the quantitative importance of anatomy variability and the lower bound. Possible deviations due to sex differences between the considered models are addressed. At last, the results are summarized and discussed in Sects. 5 and 6.

For reference, the symbols used throughout the paper and their meanings are listed in Table 1.

## Methods and models

### Finite element based method

The thermodynamical TTDE method describes the corpse cooling by the heat equation

1 $$\begin{aligned} c\rho \dot{T} = \text {div} (\kappa \nabla T) \quad \text {in}\;\Omega , \end{aligned}$$

where *c* is the specific heat capacity, $\rho$ the mass density, and $\kappa$ the heat conductivity, all of them depending on the spatial position. The heat equation (1) describes the spatio-temporal temperature distribution *T*(*x*, *t*) depending on the spatial position *x* within the domain $\Omega$, and on the time *t* in the time interval $[0,\infty [$.

The initial temperature distribution $T(x,t_0)$ given at time $t_0=0$ is assumed to be spatially constant and equal to the body core temperature $T_0 = 37^\circ C$. A more realistic choice for the initial temperature field can be obtained by solving the Bio-Heat-Transfer-Equation by Pennes [13]. The difference between these two choices for $T_0$ as well as the influence of internal metabolic heat generation on the cooling behavior are discussed in [6] and have been shown to be quite small. As the current study is focusing on the difference of anatomy models, the internal heat generation and the initial temperature field are considered to be homogeneous for simplicity.

**Table 2:** Mesh properties of the finite element meshes for the four original geometries CT1, CT2, MASH and FASH

	CT1	CT2	MASH	FASH
# vertices	178987	141004	218943	99983
# cells	961234	772837	1156853	526087

**Table 3:** Original geometric properties of the four models CT1, CT2, MASH and FASH

	CT1	CT2	MASH	FASH
Height [cm]	174	168	175.6	162.5
Mass [kg]	62	68	72.8	60.1

Heat transfer to the environment by conduction, convection, and radiation are modeled on the boundary $\partial \Omega$ by the Robin boundary conditions

2 $$\begin{aligned} n^T \kappa \nabla T = \gamma (T_A-T) \end{aligned}$$

with effective heat transfer coefficient $\gamma = h+4\epsilon \sigma T_A^3$. It relates the heat flux across the body’s surface to the temperature difference to the environment. Here, *n* is the unit outer normal, *h* the geometry-dependent heat transfer coefficient, $T_A$ the environmental temperature, $\epsilon$ the emissivity, and $\sigma$ the Stefan-Boltzmann constant. In the usual range of temperatures (270 K to 310 K) a linearization of the nonlinear Stefan-Boltzmann radiation term has a negligible impact on the cooling curve and hence on the TTDE.

Throughout this study, the cooling is simulated for 20 h after death on tetrahedral grids with the Kaskade 7 research code [14]. With convergence studies we made sure that the numerical space and time discretization errors are well below the inter-anatomical temperature deviations. Then, the simulated cooling curves for each geometry are extracted from the finite element solution at distinct measurement points. Each temperature curve *T* is represented as a finite seqence of real measurement values $(T_1, \ldots , T_N)$ on a finite grid of time values $(t_1, \ldots , t_N)$ which is the same for each simulation run.

**Table 4:** Original tissue properties (based on [17] and [18]) and volumes in the CT based models CT1, CT2 and human phantoms MASH and FASH

	$\kappa$	$c\rho$	CT1	CT2	MASH	FASH
Unit	W/m/K	J/K/l	l	l	l	l
Bone	0.75	2306.9	6.45	5.33	7.09	5.25
Fat	0.21	2116.0	19.11	25.49	18.28	21.78
Lungs	0.28	1971.2	2.17	0.97	4.90	3.92
Muscle	0.51	4123.0	30.53	39.09	41.89	29.01
Total in l			58.26	70.88	72.16	59.96
Surface in $m^2$			1.58	1.8	1.87	1.7

**Fig. 1:**
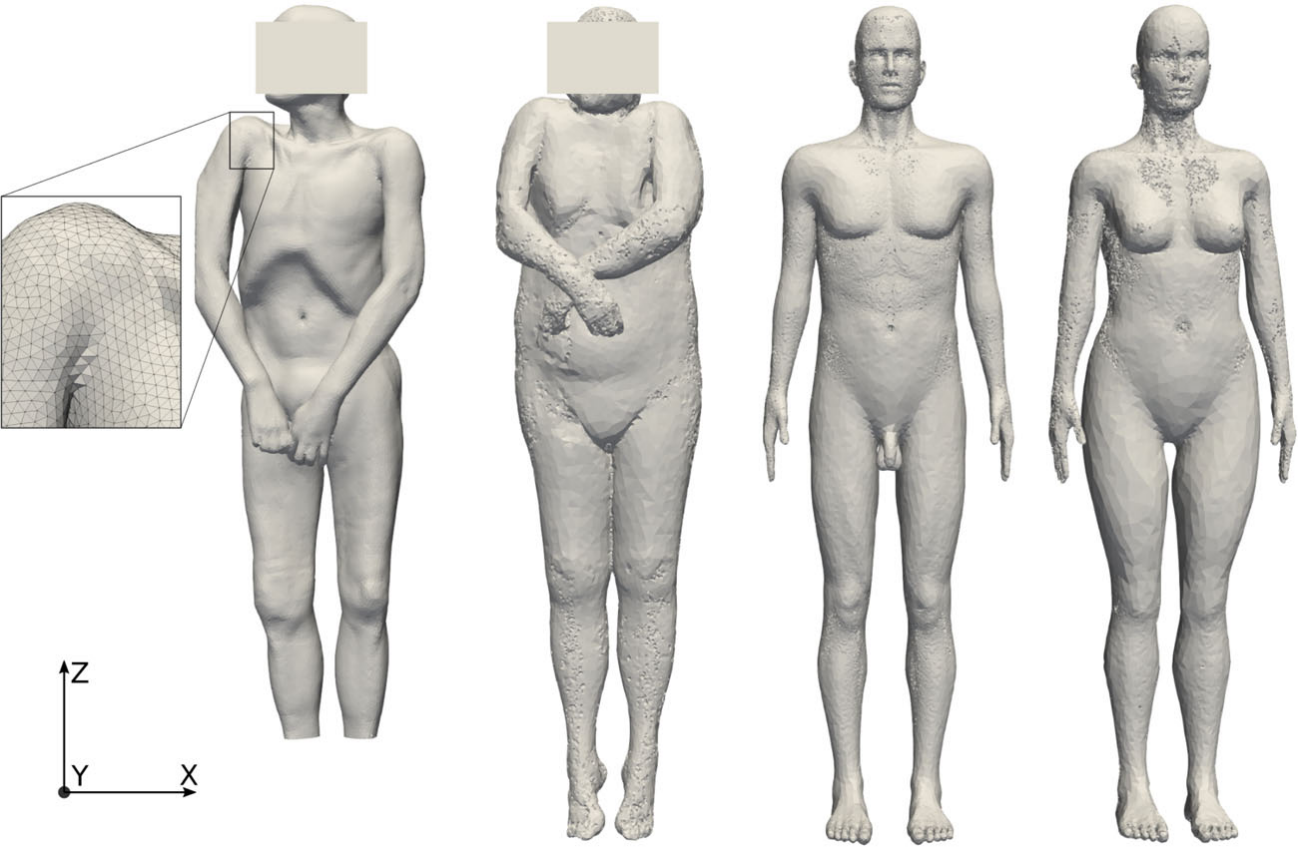
Body grids used for the study, scaled according to height and volume of reference model CT1, from left to right: CT1, CT2_sc_, MASH_sc_, FASH_sc_

### Full-body geometry models

Temperature profiles and cooling curves depend on the corpse geometry of the domain $\Omega$ and on the material parameters $\kappa$ and $c\rho$ for different tissues. Hence, for investigating the impact of geometry on the estimated time of death, we require different corpse models representing individual anatomies that are segmented into the different relevant tissue types. We use four different and independent full-body geometries:

- Two models are acquired from computed tomography (CT) data of a male and a female corpse (CT1 and CT2) [10], lying flat on an examination table,
- two human phantoms for a male and female anatomy (MASH and FASH), given as labeled voxel data sets that were created in a computer aided design tool for radiation dose planning [15], upright standing.

For the actual calculation, tetrahedral finite element meshes of sufficient resolution have been created such that each cell can be assigned a single biological tissue type [6, 16]. The mesh properties of the four geometries are shown in Table 2.

Heights and masses of CT1 and CT2 were measured directly before the CT scans and taken from [15] for phantoms MASH and FASH. Table 3 provides the original geometric properties of all used models. As distinguishing different organs of water-dominated tissue has a negligible thermal impact [6], these grids provide only bone, fat, lungs and muscle (water dominated tissue) compartments. Their properties and volumes are listed in Table 4.

Of course, the total corpse mass has a dominant impact on total heat capacity and hence the cooling curve. In order to focus on the impact of shape in contrast to size, all geometries are scaled to the dimensions of a reference model. We choose the CT1 model with its original height *L* of 1.74 m and mass *m* of 62 kg as reference model and scale the other geometries accordingly. The scaling factors are defined similarly to [2] in the following equations (3), where $k_1$ is the linear one-dimensional scaling orthogonal to the transverse plane and $k_2$ is the linear two-dimensional scaling in the transverse plane:

3 $$\begin{aligned} k_1&= \frac{L}{L'}&k_2&= \sqrt{\frac{mL'}{m'L}} \end{aligned}$$

After calculation and application of the scaling factors for CT2, MASH and FASH, a set $M = \{ CT1, \text {CT2}_\text {sc}, \text {MASH}_\text {sc}$, $\text {FASH}_\text {sc}\}$ of four models with the same height and mass (see Fig. 1) is obtained. The resulting tissue volumes are provided in Table 5. The feet missing from the CT1 scan are far from the rectal measurement position and thus not relevant for the actual calculation.

**Table 5:** Tissue properties and volumes of the geometry models in *M*, scaled to the reference height 1.74 m and mass 62 kg

	$\kappa$	$c\rho$	CT1	$\text {CT2}_\text {sc}$	$\text {MASH}_\text {sc}$	$\text {FASH}_\text {sc}$
Unit	W/m/K	J/K/l	l	l	l	l
Bone	0.75	2306.9	6.45	4.86	6.04	5.41
Fat	0.21	2116.0	19.11	23.24	15.57	22.47
Lungs	0.28	1971.2	2.17	0.88	4.17	4.04
Muscle	0.51	4123.0	30.53	35.64	35.67	29.93
Total in l			58.26	64.62	61.45	61.85
Surface in $m^2$			1.58	1.73	1.7	1.76

**Fig. 2:**
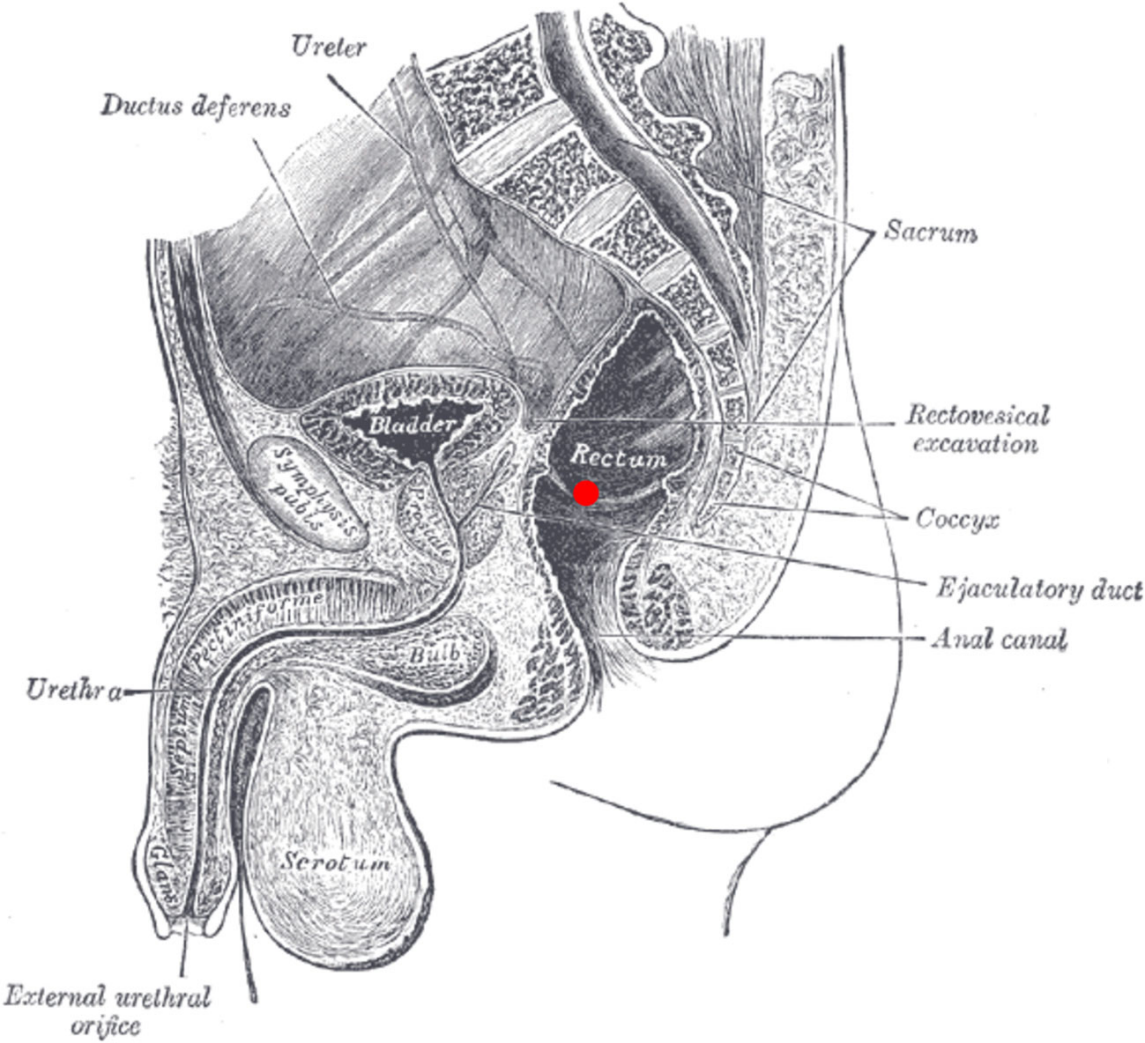
Schematic position of thermometer probe marked in red, image taken from [19]

The simulations on the tetrahedral grids were carried out for ambient temperature $T_A=20^\circ C$ and constant initial temperature $T_0=37^\circ C$ using quadratic finite elements (P2 elements) and an extrapolated implicit Euler time stepping [9].

### Selection of reference measurement positions

A single rectal temperature measurement is generally used as the value of the body core temperature. The position of the thermometer and hence the location of the chosen measurement point for simulation affects the time of death estimate. The insertion depth is not determined exactly and relies much on practical and theoretical experiences. A suitable ideal measurement position can be derived from anatomy, including forensic knowledge of the measurement locations used in practice [12]. This position in the rear part of the trunk near the lower part of the sacrum is depicted in Fig. 2.

In the virtual idealized male and female phantoms MASH and FASH, the selection of a suitable measurement position follows the anatomical template. Determining the correct measurement position in the CT-generated models, however, is more complicated. The bodies lie flat on the examination table and consequently the soft tissue is deformed to some extent. Furthermore, while human bodies all have the same structure, they differ in their local anatomical characteristics like the size of organs.

**Fig. 3:**
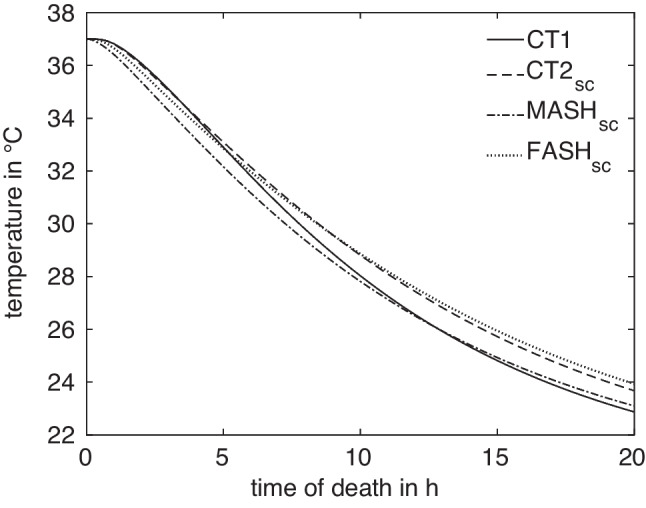
Cooling curves at the reference measurement positions $x^A_\textrm{ref}$ in the four models $A\in M$ used for simulations

**Fig. 4:**
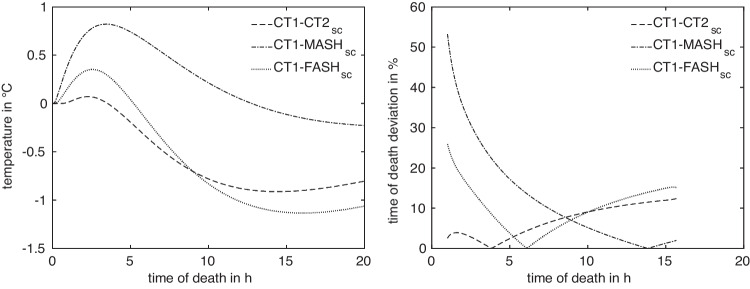
Temperature differences (left) and relative deviation $\Delta t^{A,B}_{x^A_\text {ref},x^B_\text {ref}}$ of estimated ToD (right) of $\text {CT2}_\text {sc}$, $\text {MASH}_\text {sc}$, and $\text {FASH}_\text {sc}$ models with respect to CT1 at their reference measurement positions

Reference measurement locations $x^A_\textrm{ref}$ have been chosen manually for each anatomy $A\in M$ to match the idealized anatomical template as closely as possible. Despite the care taken in selecting these reference locations, the particular choices may have an impact on the difference between cooling curves, and superimpose the effect of varying corpse geometry. This aspect is considered in more detail in Sect. 3 below.

### Quantifying cooling curve deviations

For a given anatomy $A\in M$ and measurement location *x*, the corresponding cooling curve $T^A_x$ can be computed by FE simulation, as shown in Fig. 3. All curves exhibit the expected sigmoidal shape and are monotonically decreasing, such that their inverse functions $(T^A_x)^{-1}$ can be used for estimating the times of death $t^A_x(T_m) = (T^A_x)^{-1}(T_m)$ given a temperature measurement $T_m$ at $x^A$. The vanishing derivative of $T^A_x$ for $t\rightarrow 0$ and $t\rightarrow \infty$ leads to large estimation errors for very small and very large times. We thus restrict the times considered here to the range between $t_a = 1\,\text {h}$ and $t_b = 15.5\,\text {h}$.

**Fig. 5:**
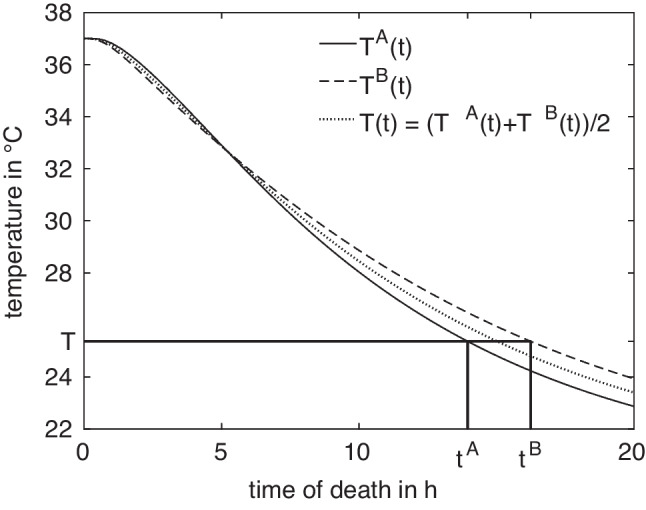
Difference in ToD between $t^A = \Delta t^A(T)$ and $t^B = \Delta t^B(T)$ for curves A and B at a certain measurement position, exemplarily evaluated for temperature $T = \frac{1}{2} \bigl (T^A(t) + T^B(t)\bigr )$ at $t=15\,\text {h}$

Anatomical differences lead to differences in the cooling curves, where the absolute temperature differences are illustrated in Fig. 4 (left). In view of the ultimate goal, i.e. the time of death estimation, differences in the resulting estimated times are of more importance for quantifying anatomy differences. Sensitivity studies suggest that ToD differences between two models *A*, *B* with measurement positions $x^A$ and $x^B$, respectively, are best captured as relative deviations

4 $$\begin{aligned} \Delta t^{A,B}_{x^A,x^B}(T_m) = 2\frac{\vert t^A_{x^A}(T_m)-t^B_{x^B}(T_m)\vert }{t^A_{x^A}(T_m)+t^B_{x^B}(T_m)}, \end{aligned}$$

relative to the mean estimated time of death. This is illustrated in Fig. 4 (right). In the absence of actual measurement values $T_m$, the general difference in model response is described by considering artificial measurement values

5 $$\begin{aligned} T^{A,B}_{x^A,x^B}(t) = \frac{1}{2} (T^A_{x^A}(t) + T^B_{x^B}(t)) \end{aligned}$$

for $t\in [t_a,t_b]$, as illustrated in Fig. 5.

In order to quantify the overall deviation of two anatomies *A*, *B* with measurement locations $x^A, x^B$, respectively, we consider the root mean squared relative deviation

6 $$\begin{aligned} D_2(A,x^A,\!B,\!x^B) \!=\! \left( \frac{1}{t_b\!-\!t_a}\int _{t_a}^{t_b}\! \Delta t^{A,B}_{x^A,x^B}(T^{A,B}_{x^A,x^B}(t))^2 \, dt \!\right) ^{\frac{1}{2}} \end{aligned}$$

and the maximum relative deviation

7 $$\begin{aligned} D_\infty (A,x^A,B,x^B) = \max _{t\in [t_a,t_b]} \Delta t^{A,B}_{x^A,x^B}(T^{A,B}_{x^A,x^B}(t)). \end{aligned}$$

These quantities can be directly interpreted as average relative ToD estimation errors, and are given in percent in the results section below. Based on these metrics, we estimate pairwise deviations $D_q$ between the anatomies in *M* and the mean total deviation

8 $$\begin{aligned} J_q^M(x^M_\textrm{ref})= & {} \frac{1}{\vert M\vert (\vert M\vert -1)}\sum _{A\ne B\in M} D_q (A,x^A_\textrm{ref},B,x^B_\textrm{ref}), \nonumber \\{} & {} q\in \{2,\infty \}, \end{aligned}$$

where $x^M_\textrm{ref} = (x^A_\textrm{ref})_{A\in M}$ is the family of reference measurement positions, and $\vert M\vert =4$ denotes the number of anatomies in the geometrical model set *M*. The mean total deviation $J_q^M(x^M_\textrm{ref})$ can be interpreted as the quantitative importance of anatomy variability on the estimated time of death. Since $D_q$ is symmetrical, the number of evaluations of $D_q$ for the calculation of $J_q^M$ can be reduced by half.

## Lower bound for impact of anatomies on ToD

### Intra- and inter-anatomical deviations

Deviations between anatomies *A* and *B*, so far equipped with reference measurement positions $x^A_\textrm{ref}$ and $x^B_\textrm{ref}$, respectively, can be due to non-corresponding measurement positions, or due to inherent differences in shape and tissue distribution (inter-anatomical deviations). Variations in the measurement position within a small neighborhood around the reference position of an anatomy have been shown to produce quantitatively significant ToD deviations [12] (intra-anatomical deviations).

While the reference measurement positions have been selected carefully based on anatomical considerations and confirmed by forensic practice, it is possible that observed ToD differences between anatomies are to some extent caused by intra-anatomical deviations. For assessing the importance of anatomical variation for the temperature-based time of death estimation it is therefore necessary to separate these two causes.

The magnitude of inter- and intra-anatomical deviations is determined by considering further measurement points around a fixed reference measurement point $x^A_\textrm{ref}$ and comparing them within the same geometry resp. across all geometries. Therefore, we create a discrete set of *n* candidate points $(x_i^M)_{i=1,\ldots ,n}$ for each anatomy. Since these locations have to be anatomically and practically reasonable, we restrict the selection to a neighborhood of the reference measurement position, i.e. $\Vert x_i^A-x^A_\textrm{ref}\Vert \le r$ for all *i*. Here, we use a radius $r=1\,\text {cm}$.

**Fig. 6:**
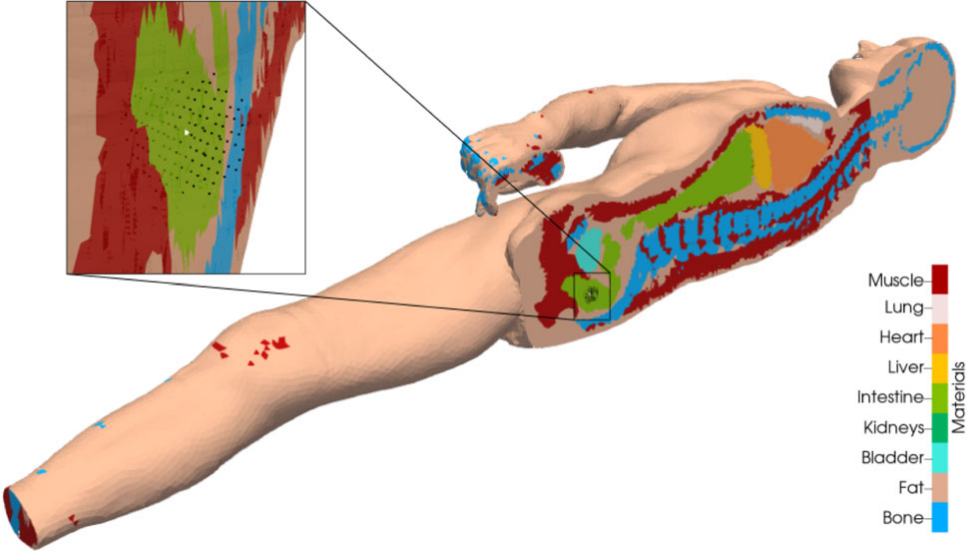
Illustration of BCC sampled additional sample positions within a ball of 1 cm radius around the original position in CT1 model

For our choice of reference measurement positions, this radius guarantees that we stay within the same tissue type in the pars ampullaris of the rectum. We then define a ball of radius *r* around the reference measurement position and generate candidate points by the body centered cubic (BCC) sampling method [20] with a grid spacing of 0.25 cm, leading to $n=137$ candidate points for each geometry, see Fig. 6.

Here, to capture intra-anatomical differences, we need to find the two measurement locations for a single anatomy *A* where the resulting cooling curve distance is maximal. Under this condition, for each anatomy $A\in M$ we maximize the deviation $D_q(A,x_i^A,A,x_j^A)$ for $q\in \{2,\infty \}$ and measurement locations $x_i^A,x_j^A$, $i,j=1,\ldots ,n$. Capturing inter-anatomical differences is, however, more intricate and will be addressed in the subsequent section.

### Optimization towards the lower bound

Due to shape and tissue distribution, the measurement positions and pairwise differences between the cooling curves are coupled in a complex way. Just subtracting the intra-anatomical deviation magnitude from the observed inter-anatomical deviation magnitude (see Fig. 4 for absolute and relative deviations) does not provide a reasonable measure of ToD differences. Instead, we eliminate the potential impact of intra-anatomical deviations and thus provide a lower bound for the ToD deviations due to inter-anatomical deviations.

We do this by determining a family $x^M_\textrm{opt} = (x^A_\textrm{opt})_{A\in M}$ of optimal measurement points such that the total deviation $J_q^M(x^M_\textrm{opt})$, with *q* either 2 or $\infty$, between all anatomies in the model set *M* is minimized. To find the family of optimal locations $x^M_\textrm{opt}$, we calculate the deviations as in equation (6) between two anatomies *A* and *B* by $D_2(A,x_i^A,B,x_j^B)$ for all candidate points $x_i^A$ and $x_j^B$. We search for the $x_i^M$ that minimize $J_q^M(x^M_i)$ such that

9 $$\begin{aligned} J_q^M(x^M_\textrm{opt}) = \min _i J_q^M(x^M_i) \end{aligned}$$

defines a lower bound on the anatomic variability. This minimization is performed in Matlab in a short amount of time by complete enumeration, leading to $137^4$ evaluations. Performing the anatomy comparison based on these optimized positions makes it possible for us to remove the differences due to the measurement point uncertainties, leaving the remaining deviations as deviations due to the anatomical differences.

**Table 6:** Inter-anatomical deviations $D_q(A,x^A_\textrm{ref},B,x^B_\textrm{ref})$ for $q=2$ (lower triangle) and $q=\infty$ (upper triangle) between cooling curves for CT1, $\text {CT2}_\text {sc}$, $\text {MASH}_\text {sc}$, and $\text {FASH}_\text {sc}$ at reference measurement positions in percent (%)

$D_2 \backslash D_\infty$	CT1	$\text {CT2}_\text {sc}$	$\text {MASH}_\text {sc}$	$\text {FASH}_\text {sc}$
CT1		12.4	53.2	25.9
$\text {CT2}_\text {sc}$	7.9		5.0	22.6
$\text {MASH}_\text {sc}$	17.4	20.1		28.1
$\text {FASH}_\text {sc}$	11.3	6.9	15.6

**Table 7:** Quantitative importance of anatomy variability $J_q^M(x^M_\textrm{ref})$ for the family of reference positions $x^M_\textrm{ref}$ in percent (%)

$J_2^M(x^M_\textrm{ref})$	$J_\infty ^M(x^M_\textrm{ref})$
13.2	32.0

## Results

### Inter-anatomical ToD deviations for reference measurement positions

First, we investigate pairwise inter-anatomical deviations using their reference measurement positions $x^M_\textrm{ref}$, as described in Sect. 2.4, and provide the results in Table 6. Both the mean relative time differences and the maximum relative time differences are provided, in the lower and upper triangular part of the table, respectively. For convenience, the values are given in percent, such that a deviation of $20\%$ for a time of death of 5 h means a deviation of 1 h, while for a time of death of 10 h it stands for 2 h absolute time difference.

Note that the values can be interpreted directly as relative ToD estimation errors, suggesting that anatomical variation can have a considerable impact of more than 20% on the accuracy of time of death estimation. The quantitative importance of anatomy variability $J_q^M(x^M_\textrm{ref})$ for the family of reference positions for all models *M* in average is listed in Table 7.

### Intra-anatomical ToD deviations

**Fig. 7:**
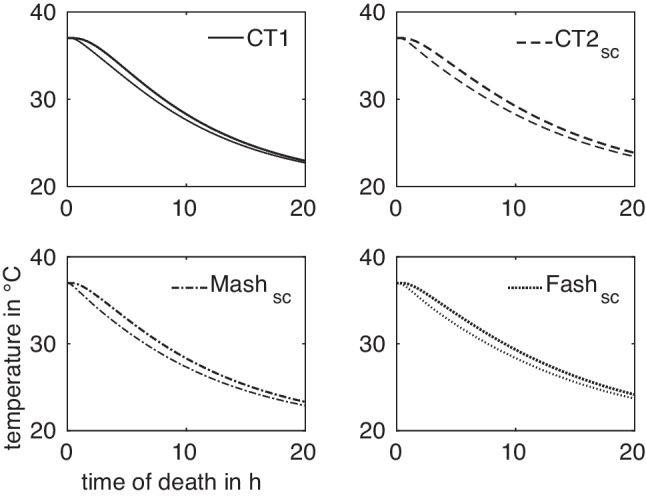
Two intra-anatomical cooling curves with maximum deviation regarding $D_2$ for the four models $A\in M$

**Fig. 8:**
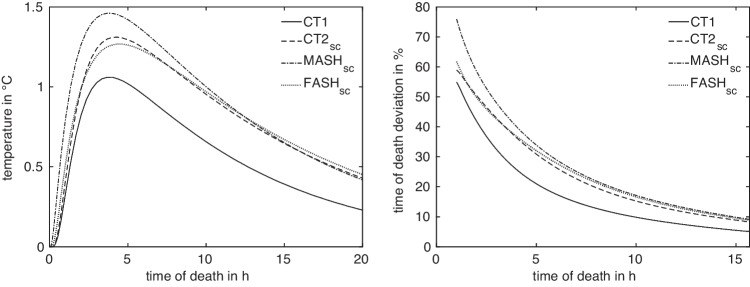
Temperature differences (left) and relative deviation $\Delta t^{A,A}_{x^A_i,x^A_j}$ of maximum intra-anatomical estimated ToD (right) of CT1, $\text {CT2}_\text {sc}$, $\text {MASH}_\text {sc}$ and $\text {FASH}_\text {sc}$ models

Next we study the intra-anatomical deviations by finding two measurement locations $x_i,x_j$ in a certain ball around the reference measurement location such as to maximize the distance of the resulting cooling curves as described in Sect. 2.4. We do this for all four anatomies of the set *M*, illustrate the results in Figs. 7 and 8 and provide the values of maximum intra-anatomical deviations $\max _{i,j}D_q(A,x_i^A,A,x_j^A)$ for $q\in \{2,\infty \}$ in Table 8. They confirm, on a larger set of anatomies, a selection of the results given in [12].

While inter-anatomical variation has an impact of more than 20% on the accuracy of time-of-death estimates, the impact in the case of intra-anatomical variation due to varying measurement locations is slightly higher in the range of 20% to 30%. However, it must be noted that the maximum values $D_\infty$ for all models are reached at the beginning of the cooling process, due to the maximization being carried out with respect to the relative deviation (6), which gives a higher importance to deviations that take place at the beginning and the very end of the cooling process.

### Lower bound on inter-anatomical ToD deviations

**Fig. 9:**
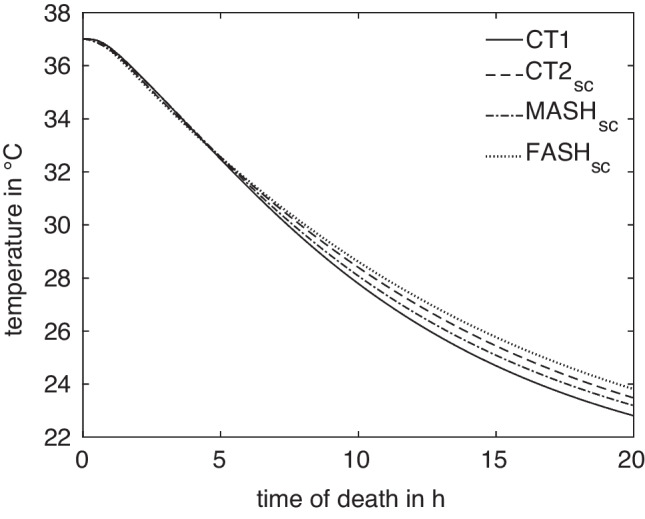
Cooling curves at the optimized positions $x^A_\textrm{opt}$ in the four models $A\in M$ used for simulations

After optimization of the reference positions $x^A_\textrm{ref}$ for all models *M* (see Sect. 3) by means of the root mean squared relative deviation $D_2$ in (6), we end up with the four overall best fit cooling curves. Figure 9 shows the four cooling curves at their optimized positions *x* and the according coordinates in the scaled models *M* are listed in table 12.

**Table 8:** Maximum intra-anatomical deviations $$\max _{i,j}D_q(A,x_i^A,A$$, $$x_j^A)$$ for $$q\in \{2,\infty \}$$ for all $$A\in M$$ in percent (%)

*q*	CT1	$\text {CT2}_\text {sc}$	$\text {MASH}_\text {sc}$	$\text {FASH}_\text {sc}$
2	21.1	27.6	31.6	28.3
$\infty$	55.6	59.9	76.4	64.3

**Fig. 10:**
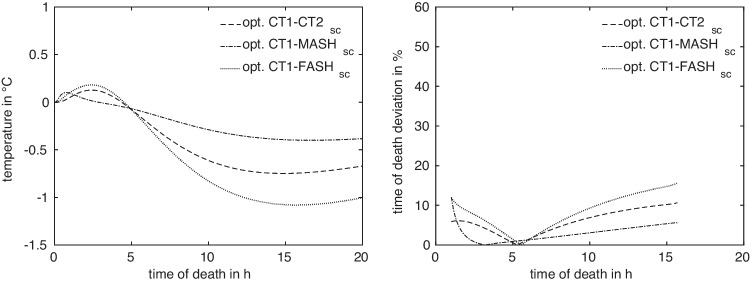
Temperature differences (left) and relative deviation $\Delta t^{A,B}_{x^A_\textrm{opt},x^B_\textrm{opt}}$ of estimated ToD (right) of $\text {CT2}_\text {sc}$, $\text {MASH}_\text {sc}$, and $\text {FASH}_\text {sc}$ models with respect to CT1 at their optimized measurement positions

The remaining deviations in the cooling curves can not be explained by an inappropriate choice of measurement location and are therefore due to anatomical differences only. Absolute temperature differences and relative deviations of ToD are shown as an example with respect to the CT1 model in Fig. 10.

Pairwise inter-anatomical deviations using the optimized measurement positions are given in Table 9. Again, both the mean and maximum relative time differences are provided. The lower and upper triangular part of the table shows the deviations measured in $D_2$ and $D_\infty$, respectively. After optimization, the anatomical variation has an effect of less than 10% on the accuracy of the TDE, indicating a somewhat smaller impact than the variation of measurement location. The mean quantitative importance of anatomy variability $J_q^M(x^M_\textrm{opt})$ for the family of optimized positions is listed in Table 10.

The actual distance between the points $x^A_\textrm{ref}$ and $x^A_\textrm{opt}$ is between 0.8 cm and 1.2 cm for all models. Since the BCC sampling does not sample the ball with radius $r=1\,\text {cm}$ exactly, there are some boundary points with a distance slightly above 1 cm. The offsets are presented in Table 13 for each anatomical plane, as well as the total offset.

### Sex differences

Another interesting aspect emerges when investigating the relative deviations $D_2$ in terms of biological sexes after optimization of the reference measurement positions in Table 9. Looking at the values for models of the same biological sex compared to the values for different sexes, we find that the values for same sexes are each the lowest in terms of the root mean squared relative deviations $D_2$ significant to us, indicating the least anatomical variation. This becomes even clearer when the individual inter-anatomical minimum between each two models is calculated, as shown in Table 14. Here, the minimum is also attained for $D_2$ for models of same sex. We point out, however, that the number of considered anatomies is not sufficient to draw significant quantitative conclusions, but is rather an indication of sex being correlated with estimated time of death.

## Discussion

Simulations carried out in advance have shown that a mere geometric scaling of different models onto each other is not sufficient to simulate the same cooling behavior. While it is possible to achieve the same cooling behavior in the simulation by scaling for simple geometries such as cubes made of a single material, significant deviations do already occur in an ideal cooling scenario for only slightly more complex geometries and heterogeneous materials.

**Table 9:** Inter-anatomical deviations $D_q(A,x^A_\textrm{opt},B,x^B_\textrm{opt})$ for $q=2$ (lower triangle) and $q=\infty$ (upper triangle) between cooling curves for CT1, $\text {CT2}_\text {sc}$, $\text {MASH}_\text {sc}$, and $\text {FASH}_\text {sc}$ at optimized measurement positions in percent (%)

$D_2 \backslash D_\infty$	CT1	$\text {CT2}_\text {sc}$	$\text {MASH}_\text {sc}$	$\text {FASH}_\text {sc}$
CT1		10.7	12.1	15.6
$\text {CT2}_\text {sc}$	6.6		5.3	5.8
$\text {MASH}_\text {sc}$	3.4	3.7		9.8
$\text {FASH}_\text {sc}$	9.4	2.8	6.3	

Significantly more intricate geometries such as human bodies have much higher deviations in shape, position and distribution of tissues and organs among each other. Therefore, it is also more difficult to determine consistent measurement points across different anatomies. Both aspects necessitate determining and classifying the differences of the anatomies in an appropriate way.

Analogously to Sect. 2.4 where we showed the differences in temperature and the relative deviation of estimated ToD for the original reference positions in Fig. 4, we investigate these differences for the optimized positions. The distance measures $D_2$ and $D_\infty$ introduced in (6) and (7) emphasize the influence of deviations at earlier times more than deviations at later times. This influence can clearly be seen after the optimization was carried out with respect to the root mean squared relative deviation $D_2$.

Comparing the temperature differences and relative deviations of estimated ToD in Fig. 4 of the four models (exemplarily with respect to CT1) at their reference measurement position with the optimized positions in Fig. 10, we notice that the differences in general are as expected reduced, especially for the early times. While we observed up to 50% relative deviation for the original measurement positions for early ToD, these deviations dropped to a value around 10% after optimization.

The distances in Table 13 indicate that the new points tend to be located more at the boundary of the considered neighborhood around the reference location. The direction of transversal, sagittal and longitudinal shifts from the reference points to the new points, on the other hand, does not seem to follow any particular pattern and is probably more dependent on individual anatomy.

**Table 10:** Quantitative importance of anatomy variability $J_q^M(x^M_\textrm{opt})$ for the family of optimized positions $x^M_\textrm{opt}$ in percent (%)

$J_2^M(x^M_\textrm{opt})$	$J_\infty ^M(x^M_\textrm{opt})$
5.4	9.9

The anatomical variability of the four different anatomies at the reference positions in Table 6 can have an individual effect of more than 20%, while the variability at the optimized positions as listed in Table 9 is less than 10%. As this study is limited to this selection of anatomies, the anatomical variability in the population and hence the impact on ToD estimation can be expected to be larger in practice. On average, up to 13% of the ToD deviations as shown in Table 7 can be attributed to the anatomical differences, which at the same time include effects due to inaccurate reference measurement positions. After optimizing the measurement positions, still about 5% of these differences as shown in Table 10 remain on average, which now represent the lower bound of adjusted differences in the anatomy.

We observe, however, a slower cooling in the long term for the female models for reference (see Fig. 3) and optimized positions (Fig. 9). In accordance with the literature, adult males tend to have a higher percentage of muscle and a lower percentage of adipose tissue than females of the same size and mass [21]. In particular, the accumulation of visceral fat, which is located near the internal organs, is more pronounced in men. However, women tend to have proportionally more subcutaneous fat [22]. We have previously shown that, among other factors, the visceral/subcutaneous coefficient and the waist to hip ratio differed significantly between the sexes in the observed study population of 35 bodies [23]. The percentage and volume accumulation of visceral fat in males tends to result in slower early central cooling of males than females. However, women have higher volume of subcutaneous fat, which covers the area near the rectal measurement point in the hip and gluteal region compared to men of the same body weight and height. They are therefore predisposed to slower long-term cooling by the effect of the subcutaneous heat barrier to the surrounding support material and air boundary layer. The vice versa effect of slower cooling in the early post mortem phase in men is apparently caused by higher central insulation in consequence of larger proportion of visceral fat.

Further, the phantoms compared to the real models have a faster cooling in the beginning of the process, meaning that their post mortem temperature plateau is shorter [24]. This is probably induced by the posture of the real bodies lying on a table such that the tissue on the back, especially fatty tissue, is displaced and gets more evenly distributed around the sample position, inducing a longer time for the temperature gradient to become established. Similar positions of the bodies should therefore also be distinguished in general.

## Conclusion

The numerical results show a clear impact of at least 5–10% of anatomical variation on the estimated time of death, even after scaling of body height and mass and removing the potential impact of measurement location. We emphasize that, given the small number of anatomies considered, which capture only a small fraction of the population’s anatomy variation, the simplified cooling scenario, and the lower bound character of removing possible intra-anatomical variation, the actual impact of anatomy variation on estimated time of death must be expected to be higher than the lower bound derived here. This, however, needs to be confirmed through further studies on a larger set of anatomies.

The results suggest that a single finite element geometry representing a standard anatomy is not sufficient for physics-based TTDE, even if scaled in body height and mass to the actual case. Instead, the specific anatomy has to be taken into account if accurate results are desired. The results also show that this cannot be compensated by defining an ideal rectal measurement location, irrespective whether this can be realized in practical casework or not.

Given that the impact of anatomy variation, while being clear, is quantitatively not dominant, we propose the use of a predefined set of template meshes, taking into consideration the distinction between female and male models to capture sex-specific tissue distribution, as well as the distinction in models of different statures and body shapes. These template grids could be generated a priori and made available in relatively coarse geometrical resolution. The actual anatomy could then be taken into account by selecting and scaling the most similar template mesh. The number and structure of such template meshes required to achieve a certain accuracy in TTDE is subject of future work.

## Appendix A. Supplementary material

**Table 11:** Original sample positions in the four models used for simulation in meters

Model	x	y	z
CT1	$-$0.0100	0.0850	0.7490
CT2_sc_	0.0140	0.1050	0.9650
MASH_sc_	0.0000	0.2150	0.8970
FASH_sc_	0.0000	0.2280	0.9080

**Table 12:** Best fit sample positions in the four models used for simulation in meters

Model	x	y	z
CT1	$-$0.0125	0.0925	0.7565
CT2_sc_	0.0115	0.1125	0.9575
MASH_sc_	0.0025	0.2075	0.8945
FASH_sc_	$-$0.0100	0.2330	0.9030

**Table 13:** Offsets of adjusted measurement positions in terms of $D_2$ from the reference points for all anatomies in *M* (in cm)

	CT1	$\text {CT2}_\text {sc}$	$\text {MASH}_\text {sc}$	$\text {FASH}_\text {sc}$
transversal	$-$0.75	0.75	0.25	0.50
sagittal	0.75	0.75	$-$0.75	0.50
longitudinal	$-$0.25	$-$0.25	0.25	$-$1.00
total	1.09	1.09	0.83	1.22

**Table 14:** Individual minimal inter-anatomical deviations $$\min _{i,j} D_q(A$$, $$x^A_i,B,x^B_j)$$ for $$q=2$$ (lower triangle) and $$q=\infty $$ (upper triangle) between each two cooling curves for CT1, $$\text {CT2}_\text {sc}$$, $$\text {MASH}_\text {sc}$$, and $$\text {FASH}_\text {sc}$$ in percent (%)

$D_2 \backslash D_\infty$	CT1	$\text {CT2}_\text {sc}$	$\text {MASH}_\text {sc}$	$\text {FASH}_\text {sc}$
CT1		10.0	4.7	15.1
$\text {CT2}_\text {sc}$	6.3		4.6	3.5
$\text {MASH}_\text {sc}$	2.5	3.5		9.0
$\text {FASH}_\text {sc}$	9.4	1.8	6.2	

## Data Availability

The individual CT scans used in the current study are not publicly available. Each single slice is linked to a DICOM-file containing experiment parameter data and sensible case information. The tetrahedral grids, the simulated cooling curves and the corresponding program code for the optimization are made available in an OSF repository (10.17605/OSF.IO/H75PW).
